# A whole system approach to childhood obesity: how a supportive environment was created in the city of Brighton and Hove, United Kingdom

**DOI:** 10.1007/s12571-023-01361-9

**Published:** 2023-04-19

**Authors:** Leah Salm, Nicholas Nisbett, Katie Cuming, Tabitha Hrynick, Alexandra Lulache, Hayley MacGregor

**Affiliations:** 1 Institute of Development Studies, University of Sussex, Brighton, UK; 2 Brighton &, Hove City Council, Brighton, UK

**Keywords:** Obesity, Systems thinking, Brighton, Inequality

## Abstract

Childhood obesity is a growing global challenge, and no country has yet reversed the upward trend in prevalence. The causes are multifaceted, spanning individual, societal, environmental, and political spheres. This makes finding solutions complex as traditional linear models of treatment and effect have proven only minimally successful or unfeasible at the population level. There is also a paucity of evidence of what works, and few examples of intervention that operate on a ‘whole systems’ level. The city of Brighton in the United Kingdom has experienced a downward trend in child obesity rates compared to national figures. The aim of this study was to explore what has led to successful change in the city. This was done through a review of local data, policy and programs, and thirteen key informant interviews with key stakeholders involved in the local food and healthy weight agenda. Our findings highlight key mechanisms that have plausibly contributed to a supportive environment for obesity reduction in Brighton according to key local policy and civil society actors. These mechanisms include; a commitment to early years intervention such as breastfeeding promotion; a supportive local political context; the ability to tailor interventions to community needs; governance structures and capacity that enable cross-sectoral collaboration; and a citywide framing of obesity solutions in the context of a ‘whole system’ approach. However, substantial inequalities persist in the city. Engaging families in areas of high deprivation and operating in an increasingly difficult context of national austerity are persistent challenges. This case study sheds light on some mechanisms of what a whole systems approach to obesity looks like in practice in a local context. This is of relevance to both policymakers and healthy weight practitioners across a spectrum of sectors who need to be engaged to tackle child obesity.

**Supplementary Information:**

The online version contains supplementary material available at 10.1007/s12571-023-01361-9.

## Introduction

Childhood overweight and obesity are rising, affecting nearly 1 in 5 (18%) school age (5–19) children globally, although there is huge variation between, and within countries (Abarca-Gómez et al., 2017; GNR, 2020). Overweight and obesity have in the past been considered an issue of high-income countries, affecting mostly the urban, middle class. Today, these conditions disproportionately affect lower socio-economic groups in high and many middle-income countries, and increasingly, also a growing number of children in low-and-middle-income countries which are progressing along a ‘nutrition transition’ (Popkin et al., 2012, 2020). The longer-term health consequences of childhood obesity are substantial and include higher risks of developing obesity and diet-related non-communicable diseases (NCDs) such as Type 2 diabetes, cardiovascular disease, and certain cancers in adulthood (WHO, 2021). Childhood obesity may also lead to cognitive, behavioural and emotional difficulties, as well as stigmatization and low educational achievement (Lee et al., 2012; Pizzi & Vroman, 2013).

Globally, knowledge on how best to tackle childhood overweight and obesity via policy, programmatic and other measures is in flux. With prevalence rising in nearly all global regions and no examples of any country reversing its obesity epidemic (Roberto et al., 2015), there is urgent need to identify what works to prevent childhood overweight and obesity. While much research has focused on downstream determinants such as diet and exercise, or on biological or (epi-) genetic factors, obesity is increasingly being considered in the context of the upstream, and much wider, social determinants of health (Marmot & Bell, 2010; Savona et al., 2019; Swinburn et al., 2011).

Obesity is therefore considered a complex and ‘wicked’ challenge. The complexity can be considered in terms of having multiple interacting aspects and the context in which they operate continuously changing because due to non-linear feedbacks, (Peters, 2014) for example between poverty related health outcomes. It is ‘wicked’ in the sense that there is no central authority to ‘fix’ it, those seeking to solve the problem may also be contributing to it, and there are multiple contested views on the problem and how it should be ‘solved’ (Peters, 2017).

Such considerations of social and other systemic determinants have led to a focus on ‘whole systems’ approaches to obesity. While there is no one definition of this, broadly, it can be conceptualised as an approach which considers multifactorial drivers,such as the ‘Obesity Systems Map’ laid out in the UK Government 2007 Foresight report which pragmatically defines an obesity system as an emergent condition, being ‘the sum of all the relevant factors and their interdependencies that determine the conditions of obesity for an individual or group of people’ (Government Office for Science, 2008). Furthermore, to act upon the complexity of obesity, a whole systems approach requires coordinated actions across a diverse range of sectors, government levels, and actors; and operates throughout the life course (Bagnall et al., 2019, Garside et al., 2010). While such approaches have been around for over a decade (Government Office for Science, 2008) and have even been promoted in national policy guidance by bodies such as Public Health England (PHE, 2019), questions remain for national and local public health authorities and others wanting to operationalise such an approach.

Public health actors may have influence or control over limited areas of local or national policy and public action, but a whole systems approach can require working across diverse sectors including housing, employment, and welfare, and working with and through others in partnership. It also implies a recognition of dynamics, and the ability to react to constant systemic flux in which relationships between individual system parts do not follow simplistic patterns of cause and effect, but depend on multiple and shifting relations. While easy to advocate for in terms of national or global policy aspirations and recommendations, more work is needed on what a ‘whole systems’ approach means in practice, including reflections on possible advantages and disadvantages, and the day-to-day challenges for public health actors and other key stakeholders.

In England, where childhood (age 10–11) obesity and overweight rates are 21% and 14.1% respectively (PHE, 2021c), the city of Brighton and Hove (hereafter referred to as Brighton)^1^ has been recognised for having lower rates of child obesity than most other cities across the UK. It has also been recognised for innovative approaches to food locally, having been among the first cities to have a food strategy, and subsequent food poverty strategy (BHFP, 2012, 2015, 2018). Indeed, it has been nationally recognised for its work on food with a Silver Award from the Sustainable Food Cities network in 2015, and was the first city in the UK to receive a Gold award in 2021.

Brighton, therefore, joins other municipalities, in offering lessons on what can be achieved via a systems approach to food and nutrition challenges. Examples include Belo Horizonte in Brazil which demonstrated longevity in its commitment to food insecurity through the initialization of urban food policy and a right-to-food approach within city governments, bolstered by a dedicated cadre of civil servants (IPES-FOOD, 2017). Amsterdam in the Netherlands pioneered a healthy weight management program which posed this challenge not only as a public health matter, but one that needed to consider across all departments through their plans, policies and day-to-day workings, further enabled through continued monitoring and evaluation, as well as the flexibility to make adjustments where necessary (IPES-Food, 2017). In New York, USA, Cohen & Ileva capture how the process of creating a more expansive food policy was achieved through efforts to address inequity in the city, through both food and nonfood areas, eg living wage, housing, and affordable healthcare policies (Cohen & Ilieva, 2020). The purpose of this study was thus to examine the case of Brighton to better understand the processes which have led to successful change with respect to childhood obesity and overweight, and to consider this within the context of related policy and programmatic interventions in the city.

This study forms part of a broader series of ‘stories of challenge’ studies undertaken by international partners which have looked at the nutritional change taking place at national and sub-national levels in five countries. It is hoped that the Brighton case will usefully contribute to understandings of what drives *downward* changes in rates of obesity and overweight in different population groups – a critical question that very few studies have addressed.

## Conceptual framing & methodology

### Background review

A background review of available literature and data was carried out in the early stages of this study. This literature and data review explored the prevalence of childhood overweight and obesity in Brighton over the past ten years, as well as contextual information about social determinants of obesity and patterns of deprivation in the city. It also laid out a cursory map of the national and local policy and program environment, describing policies, strategies, programmes and key actors and networks which are both directly and indirectly relevant to childhood obesity. This mapping drew on extensive online searching for information on the above, as well as the direct experience and involvement in this space of one of the study authors (KC). The trends and knowledge gaps highlighted in this background review formed the basis of our inquiry for further primary data collection.

### Primary data collection

Twelve key informant interviews (KIIs) involving thirteen participants were conducted (one interview involved two members of the same team).

The majority of KII participants were identified by the team to capture a broad range of perspectives across public health, community organizations, and broader food and city-based organizations in Brighton. Snowballing from interviews resulted in recommendations from participants for other potential interviewees, which is how four of the interviews were recruited. Continuous discussion within the study team and this snowballing process allowed for confidence that the key actors and a balance across a broad range of perspectives were captured in these twelve interviews.

Interviewees were working (or previously worked) with the Public Health Team in the Brighton & Hove City Council (BHCC) (n = 3), within children’s health/early years programming for the NHS (n = 2), community-based organizations (including food, weight management, physical activity and community development activities) (n = 4), and within broader local authority teams (including school transport, city planning and school meals) (n = 3).

Data collection took place virtually through semi-structured interviews lasting 45 – 90 min between November 2020 – March 2021. The interviews covered a range of topics from participants’ roles and perceptions of childhood obesity, to the identification of key actors, initiatives, moments of change and ways of working. The semi-structured approach also allowed for flexibility for participants to elaborate on their own reflections and learnings on what enabled change or a whole systems approach.

All stakeholders consented to be interviewed and were guaranteed identity confidentiality in any research outputs. To achieve this we stored the interview data securely in accordance with GDPR guidelines. Participant names and organisations are not provided and instead are replaced with their titles and broad places of work.

Ethical approval was obtained via the Institute of Development Studies’ ethical review committee, as well as through IFPRI’s Institutional Review Board (approval number PHND-19-1051). Interviews were recorded via Microsoft Teams and were transcribed verbatim.

### Data analysis

The *factors identified as driving political commitment to nutrition framework,* developed by (Baker et al., 2018), was selected to define a priori codes to analyse the interviews. Relevant framework categories were chosen and related to actors, institutions, political and societal contexts, knowledge, evidence and framing, and capacities and resources. This framework was particularly well suited to our analysis given its relatively condensed and simple way of approaching commitment to nutrition policy and capturing key policy and political economy factors.

Additional context-specific codes were identified, mostly also a priori, although some were added as we become more familiar with the material. Three transcripts were coded line by line by two researchers against the a priori codes, as well as applying open coding (Hardy, 2009) to capture Brighton specific dynamics using qualitative coding software NVivo (version 12) (QSR International Pty Ltd., 2018). Coding was compared and discussed in-depth and codes were further refined.

After this stage, an initial set of codes were defined and applied to the remaining transcripts. These included the meta categories of actors and initiatives, perceptions of Brighton, targeted approaches, partnership and coordination, capacity and leadership, and challenges and opportunities. Subcodes within each category were defined. Examples included: organisational capacity, horizontal coordination, evidence, and data systems. A full list of codes and descriptions can be found in the Supplementary Material. If additional codes were identified (these mainly related to specific initiatives, or actors), we went back over previously coded transcripts to make sure these additional codes were captured across all interviews. Each code was summarized, and a second round of grouping and condensing of codes took place. The results were shared with the wider study team for discussion and finalization.

### Validation of findings

The chronology of events was devised based on a background review of data and literature available on the topic of childhood obesity and healthy eating in Brighton, as well as key moments and initiatives identified in the KIIs. Therefore, this is not an exhaustive list of all factors that might have influenced change but rather highlights the most significant and repeated factors. This timeline was shared with participants and other stakeholders active around the healthy weight agenda for validation. Furthermore, in September 2021, we hosted a virtual validation event with some participants and additional healthy weight actors in Brighton (14 people) to share and validate findings through group discussion. This generated several of the recommendations elaborated in Table 1 of  the discussion and recommendations section below.

**Fig. 1:**
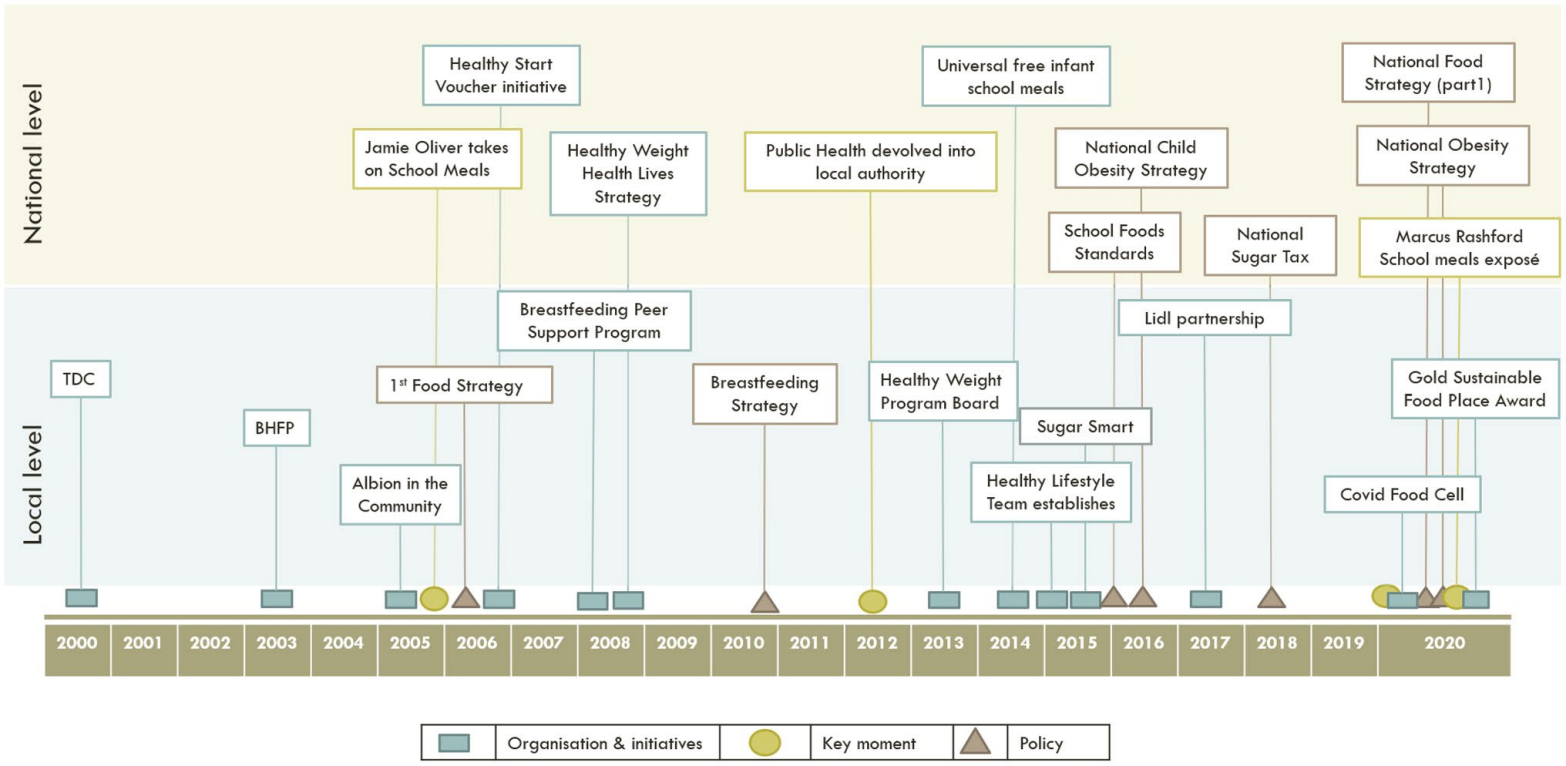
Chronology of events

Figure 1 details the chronology of important moments between 2000–2020 for organisation and initiative development (illustrated with a square icon), key moments of change (illustrated with a circle icon), and policy development (illustrated with a triangle icon). These are separated into local and national level events. Abbreviations include TDC – Trust for developing communities, BHFP – Brighton & Hove Food Partnership. This figure was created by the authors.

## Findings

### Background review of evidence health and obesity trends

**Fig. 2:**
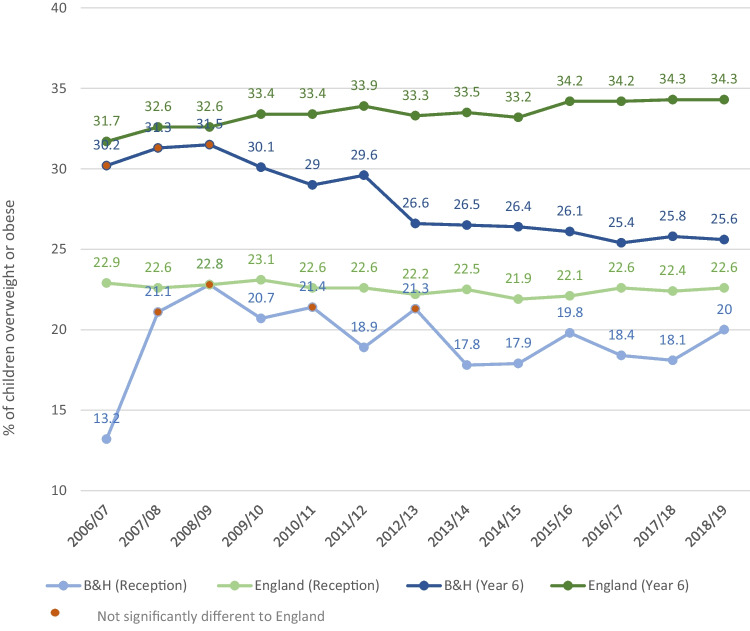
Prevalence of overweight or obesity in Brighton & Hove and England 2006–2019

Figure 2 is a line graph which illustrates the prevalence of overweight or obesity in Brighton & Hove (B&H) and in England from 2006–2019 both for reception-age children (4–5 years old) and year 6 age children (10–11 years old). This figure was created by the authors. The data were sourced from *the National Child Measurement Program Database (*PHE, 2021c*).*

The time period of interest of this study is 2008–2019. 2008 is where the first divergence from the national trend in child obesity is seen in Brighton, and 2018–19 is when this study commenced. We note that this context has changed in light of the Covid-19 pandemic. The pandemic impacts data collection and obesity rates from 2019–20 onwards. This is reflected on and contextualised in the discussion section.

Data from the National Child Measurement Programme (NCMP) shows that levels of child overweight and obesity in Brighton are lower than national and regional (South East) levels. This trend can be seen for both children in reception age, and year 6 (Fig. 2). While reception rates have been below the national average since 2012–13, year 6 rates have been consistently below the national average and declined between 2009–10 and 2012/13 during a period when the national trend was slowly rising (PHE, 2021c) (Fig. 2).

Childhood obesity in Brighton is also lower across every deprivation quintile^2^ compared to national averages. However from 2014–2019 obesity in the most deprived quintiles in Brighton was twice as high as the least deprived (25.8%, and 12.8% respectively among year 6 children, with a similar trend for reception age). The *inequalities gap* is larger than the national average gap between the most and least deprived. (PHE, 2021c).

**Fig. 3:**
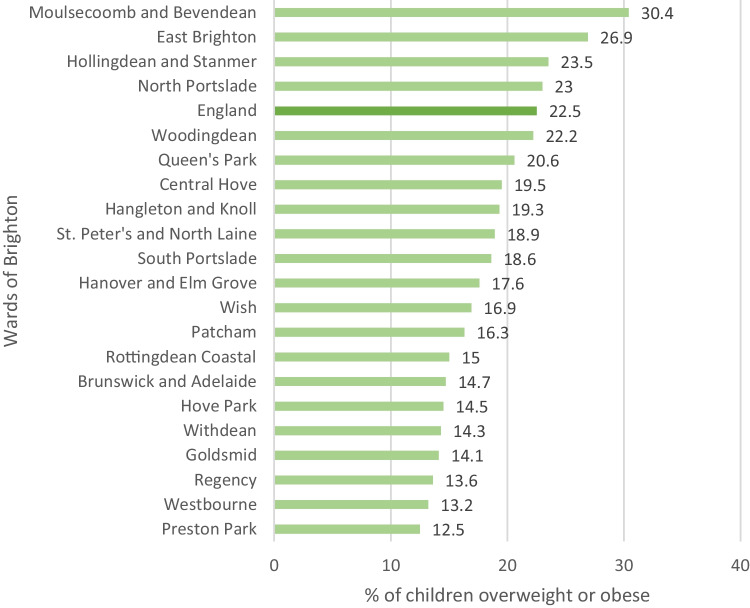
Percent of reception age children classified as overweight or obese by ward in Brighton 2016/17 – 2018/19

Families living in poverty and social deprivation are spatially clustered in Brighton, particularly towards East Brighton, Moulescoomb and Bevendean and Hollingdean and Stanmer. This concentration of deprivation is associated with a higher prevalence of obesity among children in these areas. In Moulsecoomb and Bevendean for example, overweight and obesity among reception-age children have been rising and between 2016–17 to 2018–19 the prevalence was 30.4%, significantly higher than the national average of 22.5%. This compares to just 12.5% in the ward of Preston Park, a more affluent part of the city (Fig. 3) (PHE, 2021c).

Further inequalities exist between children from ethnic minority backgrounds and white children in Brighton, with the former being more likely to be obese. This mirrors the national picture, despite Brighton having a relatively small ethnic minority population. The 5 year combined data from 2014–15 to 2018–19 show the prevalence of obesity at year 6 among white children was 12.5% compared to 19.7% and 25.5% among Asian and Black children respectively. For reception-age children, a significant difference in obesity prevalence was found between white (7.2%) and Black (15.4%) children only (PHE, 2021c).

Figure 3 is a bar chart which illustrates the percent of reception-age children (4–5 years old) classified as overweight or obese by ward in Brighton using combined three-year data from 2016/17 – 2018/19. This figure was created by the authors. The data were sourced from the *National Child Measurement Program Database* (PHE, 2021c).

Several local and national data sources are available which reflect some key health, food and social determinants of obesity in Brighton. Starting with the early years, breastfeeding rates are among the highest in the country. The city is in 5^th^ place nationally with a breastfeeding rate of 72.3% (percentage of local mothers who breastfeed at 6 to 8 weeks—measured as ‘any breastfeeding’) in 2019/20 compared to the England average of 48%. Exclusive breastfeeding prevalence at the same time point was the highest in England at 55.5% (PHE, 2021a).

When considering dietary drivers, in 2018, 69% of primary school pupils aged 8–11 years ate the recommended five or more portions of fruit and vegetables per day (a small fruit juice is also included, contributing a maximum of 1 portion per day). This falls off for secondary school children. Children from more deprived households again fare worse, with a 6 percentage point gap between the top and bottom quintiles (BHCC, 2018).

Around 31% of primary school pupils aged 8–11 years met the recommended one hour of physical activity per day in 2018. This falls to 24% for 11–14-year-olds, and 16% for 14–16-year-olds (BHCC, 2018). One study found a strong correlation between lower levels of physical activity and excess weight among children when mapped by wards (O’Sullivan et al., 2018).

Broadening the lens on drivers of child obesity, social deprivation is an important influence. In Brighton, this is captured via the national indices of deprivation for England which combines multiple indicators including income, employment, housing, health, crime, and the broader living environment. Overall, Brighton ranks within the top third of the most deprived local authorities, with 9% of Brighton’s poorest neighbourhoods being among the most deprived nationally, and falling within the bottom 10% in 2019 (BHCC, 2019). However, there is a large variation in deprivation across the city with neighbourhoods in both the top 1% least deprived and top 1% most deprived – a rare occurrence for a local authority.

The Brighton City Tracker survey reveals high levels of income poverty. In 2018, one in five adults felt they would not be able to meet basic living costs after paying for housing. Only 49% of 18–34 years olds responded they would have enough income in the coming year, while 27% of 35–54-year-olds did. The survey also revealed income disparities between white and Black, Asian and Minority Ethnic (BAME) residents, disabled residents and those without a disability (Brighton & Hove Connected, 2018).

At the time of this study, local data and secondary resources reflected a generally positive trajectory around childhood overweight and obesity, although with inequalities across gradients of social deprivation. With this as the contextual backdrop, KIIs were conducted to further investigate the nuances of the city’s overall success, yet persistent disparities in childhood overweight and obesity.

### Findings from the stakeholder interviews: supportive environment factors

#### Brighton & hove people and place characteristics

Many interviewees spoke about the ways in which Brighton’s culture, local food environment and physical landscapes could be conducive to tackling the determinants of childhood obesity and unhealthy eating. The local culture is perceived as very open and innovative despite the immediate food retail environment in the city centre which has among the highest numbers of fast-food outlets per head of population in the country (PHE, 2021d). This partly reflects the high number of day and holiday visitors using the city centre. Unhealthy food sources are partly seen as balanced out by a large number of independent food retailers promoting healthier options.

Brighton’s residents are seen as particularly open to issues around health, wellbeing and tackling inequality and as early adopters of progressive measures. There are many local activists, community groups and schemes focused on food and the environment. One interviewee reported,

*“I think it feels often as if it’s a place where things are possible, only in Brighton would kids be prepared to know that a plant-based diet is better for the environment” (Interviewee 2, Public Health Consultant, BHCC)*

The city’s natural endowments, particularly the beach and proximity to the South Downs, is seen to facilitate physical activity. ‘Active travel’ is also strongly promoted in the city and for schools by a dedicated travel team within the BHCC, which helps make the areas around schools safer for children coming to school on foot, or by bike, scooter or other forms of non-motorised transport.

#### A supportive local political context

Brighton’s pro-food and supportive local culture has translated into a local authority with strong commitments to the environment, cycling, physical activity, and breastfeeding, amongst other health-promoting factors and activities. Much of this has resulted from strong consensus-building amongst different political and local actors. Control of the local authority shifts between Green Party and Labour Party-led administrations with differing but not mutually exclusive political views, ensuring difficult political decisions become more deliverable, such as increasing restrictions on car access to the city centre. This has also enabled a strong agenda for food through the development of the city’s Food Strategy, including promoting local food production and addressing food poverty. Respondents involved with the local authority noted that it was important to have the right project at the right time, highlighting the need for timeliness when feeding into current political agendas. Several interviewees also noted political party fears about taking overtly bold action that deviate from the health and sustainability agenda, as elections are never far away. This is seen as sustaining commitments to policy.

In relation to national policy, Brighton was also seen as a positive example of good municipal coordination with national agendas, and as often going ‘above and beyond’. It was also seen to feed local information, success stories and best practices ‘upward’ to the national level, as well as ‘sideways’ to other local authorities, particularly via networks in the South East. Mentioned in particular was the influence of Brighton’s Sugar Smart initiative and the city’s voluntary sugar tax in ushering forth the national Soft Drinks Industry Levy. Further examples include Brighton’s Silver Soil Food for Life accreditation for school meals (taking food quality beyond national guidelines, to require UK sourcing of meat, the use of free-range eggs, and provision of higher quantities of fresh vegetables), alongside its aforementioned Silver and Gold ‘Sustainable Food Places’ awards. National umbrella schemes or organisations such as Sustain were seen as useful in sharing/scaling local initiatives, as were opportunities to share and learn from examples internationally, including via Brighton’s original participation in the World Health Organisation’s Healthy Cities initiative.

Brighton’s local actions were seen as having mitigated some of the damage enacted by the UK Government’s decade of austerity policies and de-funding of key services such as children’s centres. But this was coupled with a view that actions to tackle many of the broader social and political determinants of obesity and unhealthy eating still need to happen at the national level, particularly measures to do with income, employment, poverty, and a reversal of austerity-era cuts.

#### Champions for early years intervention

Brighton’s high rates of breastfeeding were attributed by participants to a longstanding commitment (15+ years) to breastfeeding promotion and reducing health inequalities through breastfeeding as a public health priority. This was seen to have been facilitated through partnership working, including between statutory bodies and the third sector, with shared goals, targets and good data. One interviewee reported,

*“there's been a very long time commitment to working together, partnership working and collaboration with different partners in the city […]. And with shared goals about what some of the wider barriers are to breastfeeding (Interviewee 1, Breastfeeding Coordinator, NHS)*

Having substantial experience working with mothers in various ways, including via peer support and targeted support to specific families, was seen as key. Particularly important has been the political will to keep funding going for Brighton’s Children’s Centres as loci of intervention and information for early years support.

#### Responding to community needs

Participants talked about a long history in Brighton of responding to community needs through tailored approaches. This was seen as linked to the city’s ethos for community grassroots initiatives and activism. Due to this, and the longstanding relationships local organisations have with residents, Brighton is often approached as a setting to trial new community interventions.

*We're also trying to look at sustainable change and working with real people on what’s actually going on in their lives, and merge academic evidence-based guidance with what people actually experience on the ground (Interviewee 13, Operations Director, community weight management organization)*

The commissioning environment (within BHCC) was seen as supportive of this approach and actively allows for the tailoring of national agenda priority items to community-level needs. Interviewees felt this was especially important because national schemes are not always designed with local needs in mind. Private funders such as the Premier League, which funds a charity running community-level sports and health activities in Brighton, were also praised for their flexibility towards project design and implementation. There is trust that those implementing have expert knowledge in what is needed and what will work at the local level.

*I get that some of it has to be top-down and I think that locally Brighton commissioners try to be as flexible as possible to allow for local sort of tailoring. That’s the thing, you’ve got to — even if it’s top-down — you’ve got to allow for communities to have a sense of their own identity and what works for them (Interviewee 6, Project manager of community development organisation)*

The strategic use of evidence facilitates community tailored approaches both for identifying communities with greater needs (for example using NCMP data to target schools in need of additional healthy weight resources), as well as for generating buy-in within communities. For example, an organisation working with communities in areas of deprivation uses local area reports generated by BHCC to start conversations with community members around health and social issues in their area, and to co-design community projects.

Partnership working also facilitates community tailored approaches. Multiple participants highlighted the response to community needs at the start of the COVID-19 pandemic as an example of this. A network of 250–300 partners, already connected through the formation of the Food Strategy, rapidly mobilized through the Food Cell (a partnership between BHCC and the Brighton & Hove Food Partnership), along with mutual aid groups to respond to community food needs. A key benefit was their pre-existing connections between each other, and with communities, and through these connections, better understood the specific needs of community members. Key individuals within these partnerships saw their role as being one of mobilising others:

*you don't [have to have] a lot of capacity, but you just need to have that personal connection, enthusiasm, and championing it, because then you release that in other people (Interviewee 5, Director of food-based community organization)*

#### Governance and capacity for partnership and cross-sectoral working

Structurally, Brighton is a unitary authority, meaning that the BHCC is solely responsible for providing all local government services. This means the Public Health Director in BHCC has significant autonomy to make decisions, leading to efficiency and potential for innovation. Furthermore, the nationwide transition of public health into local authorities (in 2012) was seen as highly advantageous to driving a broad healthy weight agenda. This has allowed for public health messaging to reach a much broader audience, and for public health interventions to be implemented across sectors that would not traditionally have been engaged. Catalysts for this include physically being in the same building, as well as a willingness and enthusiasm across departments to engage.

The formation of the Healthy Weight Program Board in 2013 was described as a key network that brings many actors (NHS, Public Health, Planning, travel, the private sector, food actors) to the table to share messaging and priorities. This cross-sector coordination feeds into a ‘whole system’ approach and allows for coordination between agendas from nutrition to physical activity, sustainability and the environment, the business environment, transport, poverty, and planning. This is a space where partnerships and collaborations can naturally form.

*But I think where a particularly strong partnership is with our public health and active for life team, I think it would be, you know, enviable for other people to know that we meet this often to discuss and there is that shared purpose. And I think that's why we end up getting quite a lot done (Interviewees 9 & 10, School Travel Advisors, BHCC)*

In addition to facilitating the coordination of agendas and resources, this structure also enables strategic thinking, both within the BHCC and externally. Examples include the designing of specific roles within Public Health that cut across sectors (such as environmental health and public health), as well as valuing and sustaining a long-term commitment to breastfeeding services. Ambitious new projects to improve the accessibility of fruit and vegetables to low-income families through collaboration between the Public Health Team, the University of Brighton, and the supermarket Lidl, are further examples of the strategic thinking that goes into local partnerships.

Successful partnership formation is achieved through the organizational capacity of the key departments, organisations, and the individuals within them. Strong working relationships, and seeing the value of partnership working is seen as a key ingredient, and allows for the sharing and prioritising of resources. The Brighton and Hove Food Partnership in particular was named a leader in facilitating partnerships across the city and as instrumental to placing ‘good food’ at the centre of the healthy weight agenda. In the face of funding and resource constraints, partnership working allows for effective coordination, and reduces duplication or wasting resources.

#### External frame resonance

Among participants, there was widespread acknowledgement that individual behaviour change alone is not enough to reduce obesity and that a broader supportive environment – including improved food environments, breastfeeding promotion, and community development to improve social determinants of health for instance – is essential.

A whole systems approach to promoting healthy weight was discussed explicitly by some participants, especially those directly involved in public health and progressing a healthy weight agenda. Participants not directly involved in forming and implementing the healthy weight agenda (such as those working in school travel, city planning and community development) have more of an implicit engagement. They acknowledged they have a role to play in creating a supportive environment and are willing to engage with Public Health and implement initiatives to facilitate healthy weight.

*We are trying to do a whole system approach towards tackling childhood obesity and healthy weight. So I think we have been ground-breaking in that respect, that we have already have that in place (Interviewee 4, Healthy Child Program Manager, NHS)*

While Brighton is seen as proactive in creating a whole system supportive environment for a healthy weight, there are persistent challenges when it comes to engaging children and families in healthy weight management programs that stem from negative perceptions of services and feelings of judgment (see Challenge Sect. 3.2.2).

Acknowledging there is still a need for targeted work with individuals and specific communities, designing messaging, and modes of delivery with communities’ needs in mind were seen as essential to resonate with people, in contrast to one-size-fits-all approaches. This creates a supportive environment to broach healthy weight that acknowledges the complexities of people’s lives and works within those bounds.

The Sugar Smart campaign was lauded as an example of successful messaging which had widespread reach across the city. Sugar Smart was developed in Brighton (and has now been scaled up nationally by Sustainable Food Places), with the aim of raising awareness about sugar in food. One participant working in public health, with experience in the dissemination of this campaign reported that it is seen as relatable to families and that, from their perspective, discussing facts about sugar allowed for openness to discuss healthy weight alongside it. Schools championed ‘sugar smart’ messaging, and this was incorporated into school meal menus. The voluntary or informed choice aspect in messaging was seen as key to inviting conversation without judging an individual’s behaviours. Sugar Smart is seen as an important factor in paving the way for Brighton’s voluntary sugar tax while supporting other city priorities like climate action through the promotion of drinking water and using reusable bottles.

*I think it was a concept that could be interpreted in lots of different ways, but it raised people’s awareness of sugar, of the importance of healthy weight as something to work towards, but I think because it wasn’t ever about “you have to do this”, but about “these are the sorts of things you could do”, I think there’s a value in that (Interviewee 2, Public Health Consultant, BHCC)*

#### Perceptions of Brighton’s areas of deprivation

Alongside the supportive environment aspects of Brighton’s response to obesity, all participants acknowledged and consistently discussed the challenge of health and obesity inequalities in the city, especially weight in areas of high deprivation.

*You have to be very careful not to get too carried away with the fact that our average is better than Southeast, our average is better than the national picture. Because it is, and loads and loads of really good work has been done, but there is still more to do, more to dig into, which particular groups are still having ongoing issues. (Interviewee 5, Director of food-based community organization)*

Both food systems and broader social determinants were seen to inhibit a healthy weight environment in these areas in comparison to elsewhere in the city, with food environments in these areas, for instance, providing less healthy food options. Decisions about food were seen by several participants to be mainly cost-driven, resulting in cheaper, lower-quality diets.

*I think the landscape changes a bit on the outskirts of Brighton, particularly in some of the areas that are in higher need. There, unfortunately, it's quite often that we have little pots of food deserts or its really difficult sometimes to find, in terms of healthy food options (Interviewee 12, Healthy Nutrition Project Officer, BHCC)*

The physical environment (including infrastructure, parks, and pavements) in these areas was also described as run down and not well maintained, discouraging children from play and making active travel such as walking and cycling difficult.

Residents living in areas of deprivation were described as having very strong community identities rather than feeling part of Brighton more broadly, and this may lead them to be less engaged with city-wide initiatives, or activities and facilities available in the city centre. Participants who work with these communities said the latter feel they ‘don’t belong’ in the city centre or the city’s surrounding natural landscapes, and that this is further compounded by limited public transport routes connecting them to these areas.

Participants working with these communities also described differences in their health-seeking behaviour compared to that of residents in more affluent areas. This included that they may feel judged or embarrassed about weight issues when engaging with health services, deterring them from seeking care. Some people in these areas were also seen to have lower expectations of their own health, often only seeking care when the deterioration in their own health impacted others:

*And then the other one is the expectation, you know, “all my aunties and uncles died in their sixties from heart attacks and had COPD [chronic obstructive pulmonary disease] so I suspect that it’s normal”. So, that notion of expectation, they expect a shorter life, and in fact if anything, most of the time when they seek intervention it’s because they’re worried about letting somebody else down (Interviewee 6, Project manager of community development organisation)*

There was a sense that while Brighton continues to grow in size and affluence, poverty rates have remained constant. Even as some prosper out of poverty, there are yet many others with high needs who take their places within areas of deprivation. The lack of affordable housing (due to the constantly shrinking supply) was repeatedly mentioned as a key challenge. Council-housed residents are often those with the highest needs, resulting in concentrations of high financial, physical health and mental health needs in areas of deprivation. Those working in these communities also said there had been a breakdown in social cohesion as newer generations of families are forced to relocate to Brighton’s outskirts.

#### Existing and planned work in areas of deprivation

All participants described how they specifically worked to close the gap in obesity prevalence in areas of high deprivation. Activities mentioned spanned health, food, physical activity, community development and active travel, delivered through targeting schools, children’s centres, food banks and community groups. Most participants acknowledged that standard channels are not sufficient and that more targeted approaches are needed to engage people in areas of high deprivation.

One participant (working in community development in areas of deprivation) explained that people should be targeted in informal spaces, by people they trust. Examples of this occurring in Brighton include providing services within the community to remove access barriers, providing informal, affordable places to socialise, such as a drop-in café attached to a food bank, and utilising Facebook groups to bring people together (which proved especially important during the COVID-19 pandemic). Participants also spoke of the importance of positive role models, such as football stars.

Projects designed to promote agency and self-efficacy were seen as key by many participants. This was evidenced through the success of Brighton’s commitment to breastfeeding peer-support services, which have built trust with and promoted self-efficacy among mothers. These services and peer-support resources have been especially reinforced in areas of deprivation. Other examples include the work of the community-led Trust for Developing Communities, in responding to community-identified needs and wants. Skills-building activities (such as ‘cooking in the community’) were provided as an example of a success story.

*I think ‘cooking in the community’ works really well because people don’t necessarily see it as a judgment on what they’re already doing, they see it as a treat, learning a new skill, learning how to cook different things they might not already cook (Interviewee 6, Project manager of community development organisation)*

Despite proactive attitudes and engagement across relevant sectors, participants lamented the lack of measures to prevent childhood obesity in the first place. Upstream social determinants of ill-health such as a lack of affordable housing, employment opportunities, and the ability of many to their meeting basic needs were seen as inevitable outcomes from a decade of the UK government’s austerity policies which have forced local authorities to make considerable cuts to spending on public services in comparison with other wealthy countries. Participants feared this would be further exacerbated in the coming years.

*It's like nearly all the resources are into interventions, not to prevention. There's a massive mismatch there and whether that's about children or adults… you have to be putting I would say five times as much for a prevention stage as you do at the intervention stage so that you're actually stopping the upstreaming. (Interviewee 5, Director of food-based community organization)*

### Findings from the stakeholder interviews: challenges

Supporting children and families already experiencing overweight and obesity to access services was a key challenge identified by many. The city’s family weight management service provider (who delivers mandated weight management programs on behalf of the city council, children are most often referred by GPs or other health and social care professionals) was seen to have the capacity to support many more families than have come forward thus far. Low numbers of referrals from health professionals to the service were repeatedly identified as a contributing factor. Participants explained that GPs, although critical contact points for families, were often reluctant to make referrals due to discomfort raising weight issues with them. Stigma experienced by parents was also seen as a deterrent to engaging with these services as feeling judged for their parenting decisions can create barriers to communicating about weight and taking action. Those working in public health also noted that discussions about healthy weight could be sensitive even within their own teams, depending on people’s personal circumstances. To help overcome these challenges, the Public Health team in the BHCC deliver training on ‘solution focused’ language to raise weight issues. The family weight management service has also started to approach community leaders such as faith leaders to support them to start conversations about weight with families.

More broadly, generating buy-in from wider city actors such as city planning officials and the private sector can be challenging given the voluntary nature of engagement. For example, the Healthy Choice Catering scheme which encourages private food caterers to offer healthier options offers voluntary accreditation, and there is no obligation to engage. Similarly, the BHCC City Planning department promotes food growing spaces in new development plans, but these are not highly prioritised and are therefore rarely implemented by developers.

Targeting schools was seen as a strategic opportunity to reach all children at once. However, participants who have worked with schools noted this could be challenging, depending on the capacities of a given school. Often, they rely on a member of staff to take ownership and champion an intervention, but this is not always possible. Furthermore, primary schools appear to host most food and physical activity interventions, while there is virtually no targeting of secondary schools. This was seen as a key challenge given the drastic change in adolescent children’s food environments as they move into secondary school, and also have increased autonomy over food decision-making.

Finally, the broader commissioning system was seen as a challenge to sustainable intervention. Funding cuts have resulted in resources being thinly stretched and prioritised to essential services leaving the capacity to deliver smaller projects hampered and lacking in the long term commitment needed for sustainable programming and service delivery.

*I just think until you work with real people and see how their lives actually look like and actually what behaviour change looks like for them, there's such a big gap between what gets released and the reality for me, "oh just eat better and exercise" its like..yeah. (Interviewee 13, Operations Director, community weight management organisation)*

## Discussion & recommendations

The causes of overweight and obesity and related NCDs are known to be multifaceted, spanning individual, social, and environmental dimensions. On a broader level, they are driven by obesogenic food and cultural environments which influence the conditions in which people live, work, travel, and acquire food (Friel et al., 2007). Socioeconomic status and forms of exclusion or discrimination are known to intersect with these environmental influences, creating inequalities in the prevalence of obesity and the distribution of associated NCDs (Kumanyika, 2019). Traditionally, public health obesity and healthy eating interventions have focused on targeting individual behaviour determinants, with mixed success (Backholer et al., 2014). In recent years there has been a shift towards recognition that broader food and social environments need to be altered to promote healthy weight in the long term (Weihrauch-Blüher & Wiegand, 2018). In the UK, successive obesity policies have focused on and have encouraged a whole-systems approach to obesity, with government guidance encouraging public health leaders in local authorities to adopt such an approach (PHE, 2019).

It was in this context that we sought to understand the factors that may explain how childhood obesity prevalence in Brighton has bucked the upward national trend to actually reduce in recent years and to consider this success through the lens of a whole systems approach. We have documented two related processes: the evolution of the city’s policies and programmes and improvements in the city’s child obesity figures. While our methodology cannot (nor does it seek to) establish clear causal pathways between these processes, we have been able to tell a detailed story of this system based on what key stakeholders have reported has worked well and what may have led to the creation of a supportive environment for obesity reduction in the city. We believe that this rich detail may provide lessons for others wanting to follow a similar approach elsewhere.

In Brighton, the following factors were identified as important parts of the city’s success by stakeholders holding key roles in designing and implementing city-wide food and public health strategies. We see the combination of factors as having created the supportive environment, and together, are greater than the sum of their parts. This allows for a system that can be responsive to a continuously changing situation and act on multiple levels. The factors include:

- A set of multi-actor, multi-sector and inclusive policy processes has helped mobilize resources, increased problem-solving capacity, promoted strong relationships with local businesses, and ensured the presence of a variety of important perspectives at the table – particularly of different types of service providers. Some stakeholders felt the structures were less important than the relationships between individuals. These relationships were enabled by cooperative dynamics within the local authority, as well as through the brokering of non-government organisations such as the Brighton & Hove Food Partnership, with strong links across both the statutory and voluntary/community sectors.
- Locally driven action was also enabled by the fact that city-level authorities had the requisite power and responsibility to mobilise. This power, devolved from the national level – particularly as embodied in the shift of responsibility for Public Health from the NHS to local authorities – was seen as allowing for considerable innovation with fewer constraints.
- Political commitment was secured from council leaders and sustained even whilst power shifted regularly between Labour Party and Green Party administrations.
- Brighton’s overall strategy was seen as supported by a national policy context favouring whole systems approaches, even though several stakeholders pointed out the challenges presented by the national context.
- Alongside political leadership, key individuals within the local authority and the city’s voluntary sector, particularly the Brighton & Hove Food Partnership, drove and maintained the city’s commitment to food. Developing a city Food Strategy was seen as key to harnessing partnership and city-wide commitment.
- While the city-wide focus was often on broader food issues (especially illustrated in Fig. 1), a sustained focus on early years and targeted school-based interventions, particularly in more deprived areas, was seen to have driven improvements amongst key age categories and target groups
- Service providers and the council’s Public Health Team and Healthy Weight Programme Board made good use of the various data sources available to them to target support, for example to individual primary schools. Where data gaps have been left by the absence of national surveys, efforts have been made to collect data locally, such as on breastfeeding prevalence.

Our findings also detail several challenges faced by local stakeholders, particularly in reaching families in Brighton’s more deprived areas. One participant running services in these communities noted at our validation meeting that while there are in fact many ongoing activities, initiatives and services available, they are not being fully utilised. People may feel that services are not suited to them or that they are stigmatising. They may also simply be unable to access them due to other social factors such as employment or housing constraints. Other stakeholders at the validation session mentioned how the national policy environment actually undermines local level efforts in some ways. A period of austerity since the 2007 recession has led to cuts in social spending, and healthcare services. These cuts are largely seen to disproportionately impact the most vulnerable members of society through higher unemployment, homelessness and poverty, with implications for mental health and food security (Stuckler et al., 2017, Marmot et al., 2021).

This study began before the COVID-19 pandemic when a downward trend in child obesity prevalence was evident. The most recent data at the national level shows obesity rates for both reception and year 6 children increased by approximately 4.5% between 2019–20 – 2020–21. This is the highest jump since the NCMP began in 2006–07 (PHE, 2021b). The latest data also reflects a widening gap between the most and least deprived areas across England. The data for Brighton from 2018/19 onwards also shows an upward trend for both reception and year 6 children. Data gathered by the Brighton & Hove Food Partnership reports that food bank usage in March 2021 remained almost four times higher than July 2019, serving 1825 families per week compared to 420 respectively (BHFP, 2021). The interviews for this study took place after the pandemic had begun, and naturally, all interviewees spoke to the very real and pressing challenges it posed in widening health inequalities. In addition to increased food bank usage, participants also mentioned issues such as children (initially) not having access to school meals or getting enough physical activity, while families lost income, and struggled with mental health challenges.

Given the clear links between obesity and deprivation, as well as the fact that obesity is a primary risk factor for covid-19, there was a heightened sense of urgency among participants to prioritise action for tackling obesity. This urgency was also evident in the national policy space where bold measures—such as the banning of promotions on junk foods and the advertising of these foods to children on television – were taken (Department of Health and Social Care, 2020). The pandemic also highlighted the importance of community engagement as a form of resilience, achieved through the power to mobilise rapidly and respond to vulnerable people’s needs.

Although it was not a specific objective of this study to produce policy recommendations, in the September 2021 validation session, stakeholders were invited to reflect on what local and national recommendations might emerge from the study to ensure sustainability in Brighton (particularly in the light of covid impacts) and replicability elsewhere. Table 1 presents these recommendations, following a group discussion and a further round of comments by participants of the validation session.

**Table 1:** Local and national recommendations developed during the validation session

	**Early years**	**School setting & targeting children**	**Addressing inequalities**	**Whole system approaches**	**Workplace**
**Local-level**	Accessing & engaging with parents in areas of deprivation. Requires sensitivity & understanding of the complexity of people’s lives	Targeting school food environments, especially secondary schools (including food businesses around schools)	Bridging services with those who need access the most. Requires more structure & commitment to include Black, Asian and Minority Ethnic communities & other minorities, including understanding complex needs	Long-term commitment is valued and maintained. Requires appropriate and flexible funding &timelines	Information around services, local support that is in situ, which is streamlined with, and reiterates early years messaging.
	Proactive follow up & support provided by trained professionals		Co-production of understanding complex needs/ family needs	Ensuring principles for creating healthy weight environments are embedded in planning/transport policy.	
**National level**	Reinstate national infant feeding survey & develop national breastfeeding strategy. This should link to healthy feeding & eating more broadly & requires more joined-up thinking across the life course	Ban Junk Food advertising to children including online	Monitoring how cuts to the benefits system impact inequalities & food security. Improve social safety nets	Implement current national food strategy & obesity strategy recommendations. Including collectively pushing for the food strategy white paper to insure implementation.	
				Conflicts of interest need to be considered in whole systems approaches, with appropriate rules of engagement enforced.	
				Promote the development of local food strategies in other areas – considered a key starting point in taking a whole system approach.	

Systemic approaches to obesity reduction remain few and far between. A recent systematic review finds evidence on city-wide approaches to obesity reduction largely piecemeal, and heavily biased towards North America. The authors call for a combination of educational, fiscal, regulatory and environmental approaches to address obesity in cities (Danielli et al., 2021), which we believe Brighton to come close to. Elements of Brighton’s story have been reflected in other parts of the world, although relating to other forms of malnutrition such as stunting (low height for age). For example, the state of Chhattisgarh in India saw a decline in childhood stunting from 52.9% to 37.6%, while neighbouring areas did not. Features of this success include ‘a shared vision for impact’, political stability and functioning administrative systems, as well as coordination across civil society and development partners, and community mobilisation (Kohli et al., 2020). Similarly, a synthesis across five countries (Nepal, Ethiopia, Peru, Kyrgyz Republic, Senegal) that have experienced reduced stunting at a national scale reports the importance of immediate targeted intervention, as well as wider health systems and cross-sectoral action such as investment in reproductive health, poverty alleviation, universal education, food pricing policies, data system strengthening and sustainable financing for nutrition (Bhutta et al., 2020). Furthermore, several of the identified actions and ways of working in Brighton are also reflected in recent food systems policy recommendations to deliver healthier diets for all, particularly concerning actions around school meals, business incentives, taxation and nutrition education (Hawkes et al., 2020).

A limitation of this work was our lack of capacity to include the perspectives of those experiencing healthy weight challenges. This would no doubt provide an additional, very important, perspective about the lived experience of navigating child obesity at a household level and further contextualise our findings of Brighton’s community tailored approaches. We recommend further research on this as an important complement to knowledge in this area. Additionally, a member of this research team is a Public Health Consultant with the BHCC and was also a participant in the KIIs of this study. Given the author's key insights and knowledge as a public health practitioner, this is considered a valuable addition to the KII information gathered. Furthermore, while we made every effort to include a diversity of stakeholders that are directly related to or adjacent to the healthy weight agenda, through our own knowledge and networks, and snowballing from interviewees, there is always a risk that we are missing other or opposing view points. Due to the exploratory associations (rather than causal links) made in this work, it is not considered to bias our findings.

## Conclusion

This study set out to explain the story of change with regard to child overweight and obesity in the city of Brighton, UK. Trends in these outcomes have held static or have even in some years declined, particularly among children aged 10–11. Brighton has therefore bucked national trends of *rising* prevalence, also occurring in many other countries worldwide.

Evidence from stakeholder interviews, literature and data reviews support a picture of a city where a ‘whole systems’ approach to obesity across different municipal, state, voluntary and private sector domains is thriving. This approach has been actively promoted by key individuals and coalitions of actors within the public health and other teams within the local authority itself, and within key voluntary organisations who have worked to ensure change across the local food system, particularly the Brighton & Hove Food Partnership.

Our study design leaves us unable to conclude a causal relationship between this approach and the comparatively positive picture of child overweight and obesity, even though we find it plausible. Another part of Brighton’s story relates to the significant challenges it still faces. These include meeting the needs of residents experiencing high poverty and deprivation, managing the local fallout of a decade of UK government reductions in public spending, and contending with the continuing challenge Covid-19 poses to health inequalities in the city. Brighton has its own unique attributes (as any local context does) which limit the direct replicability of the city’s approach elsewhere. Nevertheless, this case study offers a view of factors, mechanisms, and lessons – developed with the participation of many key actors in the city’s public health and food landscape – in fostering partnerships for obesity reduction through a whole systems approach.

## Supplementary Information

Below is the link to the electronic supplementary material.

Supplementary file1 (DOCX 35 KB)

## Data Availability

The obesity prevalence data to support these findings are opening available from the Public Health England National Child Measurement Database available here: https://digital.nhs.uk/services/national-child-measurementprogramme/. Anonymized data from key informant interviews are available upon reasonable request from the corresponding author (LS).

## Footnotes

1 Brighton and Hove constitute one unitary local authority. The two entities joined in city status in 1997, although they are often referred to as Brighton. The data presented refer to all boundaries of Brighton and Hove.

2 Deprivation is measured via an Index of Multiple Deprivations which includes multiple indicators related to income, employment, housing, health, crime, and the broader living environment. There are 5 quintiles of deprivation for which obesity data is available publicly raging from the most to least deprived. The quintile breakdown is only available for obesity (including severe obesity) data, and not for overweight and obesity combined.
