# Supplementary material for: A whole system approach to childhood obesity: how a supportive environment was created in the city of Brighton and Hove, United Kingdom
Source: Food Secur. 2023 Apr 19:1–17. Online ahead of print. doi: 10.1007/s12571-023-01361-9 (PMC10113721; doi:10.1007/s12571-023-01361-9)
Supplement: Supplementary file 1 — Supplementary file1 (DOCX 35 KB) [file 12571_2023_1361_MOESM1_ESM.docx]

## Supplementary Material

A whole system approach to childhood obesity: how an enabling environment was created in the city of Brighton and Hove, United Kingdom

Leah Salm*, Nicholas Nisbett, Katie Cuming, Tabitha Hrynick, Alexandra Lulache, Hayley MacGregor

*corresponding author – Institute of Development Studies, Brighton, UK. [l.salm@ids.ac.uk](mailto:l.salm@ids.ac.uk)

Journal - Food Security

## Table 1: Coding tree, source, and description

| Category | Codes / coding source | Code Description in Brighton context |
| --- | --- | --- |
| 1 Actors and initiatives | **Actors**  Open coding - Brighton specific | Actors involved in healthy weight agenda including framing problem, generating evidence, implementation of initiatives both specific and adjacent to healthy weight agenda. |
|  | **Initiatives/ projects**  Open coding - Brighton specific | Key initiatives, projects, activities that participants have worked on or are aware of as playing a role within the healthy weight agenda. |
| 2 Perception of Brighton | **Societal cultural norms**   A priori - Baker FW | Societal conditions/cultural norms in Brighton broadly speaking and related to healthy weight behaviours or perceptions. Includes long terms phenomena and short-term processes and can both present opportunities and impediments to healthy weight agenda. |
|  | **Perception of physical** **/food /infrastructure environment**  Open coding - Brighton specific | Brighton specific descriptions of the physical environment (green spaces, South Downes), food environment (vendors, availability of food), and infrastructure (roads, safety, public and active travel options) as well as how people interact with these environments. |
|  | **Perception of healthy weight**  Open coding - Brighton specific | Opinions on child healthy weight/ obesity trends across the city, including changes over time, and key influences. |
|  | **Perception of breastfeeding**  Open coding - Brighton specific | Perception of attitudes and norms within society in Brighton regarding breastfeeding, as well as drivers of these behaviours/ norms. |
|  | **COVID-19 impacts**  Open coding - Brighton specific | Perception of the impacts of COVID-19 across the city and residents including vulnerabilities and impacts on healthy weight behaviours. |
| 3 Targeted Approaches | **Targeting early years**  Open coding - Brighton specific | Perceptions of how early years have been targeted through intervention, including breastfeeding promotion, children centres etc. Facilitators and challenges to early-year intervention in Brighton. |
|  | **Targeting inequalities**  Open coding - Brighton specific | Descriptions of initiatives that aim to specifically target inequalities both directly related to healthy weight and broader social determinants of health. Facilitators and challenges to targeting inequalities in Brighton. |
|  | **Community tailored approach**  Open coding - Brighton specific | Evidence of tailoring of interventions to specific community needs/ taking community led approach to design interventions. Facilitators and challenges to community tailored approaches. |
|  | **Targeting schools**  Open coding - Brighton specific | Initiatives that target the school setting specifically in relation to healthy weight activities. Benefits and challenges of a school-setting based approach |
|  | **Key moments**  Open coding - Brighton specific | Key moments of change, or initiatives that have shaped the healthy weight agenda. |
|  | **Covid specific projects**  Open coding - Brighton specific | Identification of how interventions related to the healthy weight agenda have adapted to COVID-19 restrictions, as well as identification of new initiatives that have stemmed from the crisis to meet needs. Facilitators and challenges to COVID-19 operations. |
|  | whole system  Open coding - Brighton specific | Description of ‘whole system’ ways of operating in relation to healthy weight agenda. Code reserved for specific use of this terminology. |
| 4 Partnership and Coordination | **Partnerships and coalitions**  A priori - Baker FW | Partnerships and coalitions within the actor network working forwards healthy weight goals. Includes the modes through which these partnerships operate and are sustained. |
|  | **Private Sector Engagement**  Open coding - Brighton specific | Mentions of private sector engagements, the benefits, and challenges of these engagements. |
|  | **Vertical coordination**  A priori - Baker FW | The degree to which healthy weight policies and initiatives are effectively coordinated,  implemented and monitored across levels of governance. Particularly regarding how national policy is translated to the local Brighton context. Also including the relationships and flow of information across governance levels. |
|  | **Horizontal coordination (across sectors)**  A priori - Baker FW | Evidence of coordination laterally across sectors, including synergies with other agendas, shared goal/ target setting. Facilitators and challenges to horizonal coordination. |
|  | **Internal frame alignment**  A priori - Baker FW | Evidence of unification within the healthy weight actor network around common understanding of healthy weight challenges, goals, and solutions. Mentioned benefits, facilitators, and challenges to internal frame alignment. |
|  | **External frame resonance**  A priori - Baker FW | Public portrayals (frames) used within the actor network related to overweight/ obesity cause, responsibility, severity used to resonate with external audiences in Brighton or nationally. Includes modes of delivery of messaging, facilitators, and challenges to external frame resonance. |
| 5 Capacity and Leadership | **Organizational capacity**  A priori - Baker FW | Perceptions of the degree to which healthy weight actors possess the technical skills, knowledge, administrative and human resource capacity, as well as management and logistical capacity required to mobilise the healthy weight agenda. Factors that facilitate or inhibit organizational capacity. |
|  | **Financial capacity**  A priori - Baker FW | Perception of the degree to which budgetary commitments and financing systems support and incentivise the healthy weight agenda. Factors that facilitate or inhibit financial capacity. |
|  | **Evidence and data systems**  A priori - Baker FW | The use of evidence and data systems (or lack thereof) in identifying healthy weight trends, gaps in knowledge and role of evidence in decision making. |
|  | **Ideology and institutional norms**  A priori - Baker FW | Identification of the entrenched belief systems, values, and practices of healthy weight actors as well as broader political actors. How these ideologies and norms influence the healthy weight agenda. |
|  | **Legislative, regulation and policy FWs**  A priori - Baker FW | Identification of strategies/ partnership plans/ local/national policies that provide a mandate and authority to act, therefore can be seen as formal arrangements that influence the healthy weight agenda. How these are developed and/ or received by local implementers. |
|  | **Strength and leadership**  A priori - Baker FW | Presence of committed and politically savvy actors (both individuals or organisations), within or outside of government with a commitment to driving the healthy weight agenda. |
|  | **Champions**  Open coding - Brighton specific | Individuals identified who are seen to mobilise resources around the healthy weight agenda, and sustain commitment to important issues. Champions can occur at any level from community to policy level. |
|  | **Strategic thinking**  Open coding - Brighton specific | Examples of actors having a strategic approach within the healthy weight environment, may include a clear direction of action, forecasting/ setting strategic priorities for the future, or creating innovative plans to channel resources. |
|  | **Supportive political contexts**  A priori - Baker FW | Overarching political context (both local Brighton and national) and the extent to which this helps or hinders the healthy weight agenda, including influence on commitment, decision making, resourcing. |
| 6 Challenges and opportunities | **Challenges**  Open coding - Brighton specific | Future challenges in the pursuit of the healthy weight agenda. |
|  | **Opportunities**  Open coding - Brighton specific | Future opportunities in the pursuit of the healthy weight agenda. |
